# Lifestyle counselling – a long-term commitment based on partnership

**DOI:** 10.1186/s12875-022-01642-w

**Published:** 2022-03-01

**Authors:** Lena Lönnberg, Mattias Damberg, Åsa Revenäs

**Affiliations:** 1 Center for Clinical Research, County of Västmanland, Uppsala University, Västerås, Sweden; 2 Department of Public Health and Caring Sciences; Family Medicine and Preventive Medicine, Uppsala University, Uppsala, Sweden; 3 School of Health, Care and Social Welfare, Division of Physiotherapy, Mälardalen University, Västerås, Sweden; 4 County of Västmanland, Orthopedic Clinic, Västerås Hospital, Västerås, Sweden

**Keywords:** Qualitative research, Healthy lifestyle, Counselling, Primary health care, Diabetes mellitus, Type 2, Hypertension

## Abstract

**Background:**

Lifestyle habits are important factors in the development of non-communicable diseases. Different ways of providing counselling in primary care to promote healthier lifestyle habits have been launched and evaluated in recent years. It is important to provide an insight into what makes lifestyle counselling useful for patients and healthcare providers.

**Objective:**

The overall aim of this study was to explore patients´ and community health nurses´(CHNs) experiences of lifestyle counselling in primary care to support healthier lifestyle habits.

**Methods:**

Patients and CHNs were interviewed, face to face. Sixteen patients (eight men, eight women, aged 51–75 years) diagnosed with hypertension or type 2 diabetes mellitus and three CHNs participated. Data material was analysed with qualitative content analysis to explore the participants experiences of lifestyle counselling.

**Results:**

The theme demonstrates that lifestyle counselling is a long-term commitment based on partnership between patients and CHNs. Five categories describe this partnership: respect and mutual interest, understanding of illness, measurements and goal setting, long-term support, and a structure to support counselling within the primary care unit.

**Conclusion:**

The results from this study are consistent with and add to previous understanding of how lifestyle counselling can be performed successfully in the context of primary health care. The results emphasize that lifestyle counselling should encompass a partnership based on mutual respect, recognition of the patient as the expert on his/her current life situation, and the need for both parties to engage in the process of lifestyle change.

**Practice implications:**

A structured lifestyle program with five counselling sessions within primary care was experienced as helpful for enhanced lifestyle habits and considered to be feasible by both patients and CHNs.

## Introduction

According to a systematic analysis for the Global Burden of Disease study 2019, the leading risk factors for attributable deaths were high systolic blood pressure, followed by tobacco use and high fasting plasma glucose concentration and all but one of the ten leading causes of death in high-income countries were related to conditions in which lifestyle habits have an important influence. In addition, the combined burden of diet quality, physical inactivity, and high body mass index was about 12% (CI 95%: 9.6–14.5) of all disability-adjusted life years in 2019 [1–3].

Both national and international guidelines on cardiovascular (CV) prevention, diabetes care and cancer prevention emphasize the importance of a healthy lifestyle to halting or slowing the global pandemic of lifestyle-related diseases [4–10]. They underline that the health-care system should provide patient education and personalized support to enable patients to self-manage their condition and increase self-efficacy for maintaining healthier lifestyle habits [4, 5, 9].

Different ways of providing counselling to promote healthy lifestyle habits have been launched and evaluated in recent years. A systematic review of methods for behavioural counselling to promote healthier lifestyle habits to prevent CV events reported wide diversity in the interventions tested. However, despite the diversity, the programmes were found to have positive effects on metabolic risk factors [11].

Interventions to initiate behaviour change are often complex and include different components. Michie et al. has described these components, or behaviour change techniques (BCTs), as ‘a replicable component of an intervention designed to alter or redirect causal processes that regulate behaviour; that is a technique proposed to be an “active ingredient” (e.g. behaviour or outcome goals, feedback, self-monitoring and reinforcement)’ [12]. In recent years, the approaches to managing chronic disease have shifted from the traditional provider–patient relationship to a paradigm in which the person with a chronic condition plays a key role, in partnership with their health-care providers [13–15].

To enhance the care of patients recently diagnosed with hypertension, type 2 diabetes mellitus (T2DM) or impaired glucose tolerance and to address their future risk for cardiovascular disease (CVD), we started a one-year, structured lifestyle programme at a primary care unit in Västerås, Sweden. The effect on lifestyle habits and CV risk factors has been evaluated earlier by Lonnberg et al. as a part of an on-going PhD study [16, 17]. The programme was designed to be included in everyday clinical practice, which would allow delivery of the programme for patients in their ordinary primary care setting with patients and community health nurses (CHNs) at the center of counselling to promote healthier lifestyle habits.

Despite the growing evidence of the effectiveness of lifestyle interventions in reducing CV risk, fewer scientific evaluations have focused on lifestyle interventions delivered in Swedish primary care and in clinical practice in general. The National Board of Health and Welfare in Sweden has noted a need for more structured treatment based on National Guidelines for treatment and prevention of unhealthy lifestyle and secondly, more can be revealed on how patients and CHNs experience counselling to promote lifestyle change [18]. The involvement of patients is essential for increasing their control over lifestyle habits and health. Therefore, it is important to understand the patients’ experiences of participating in a lifestyle programme. Accordingly, there is also a need to explore the CHNs’ perspectives of the same issues to identify the knowledge, skills, and attitudes needed to deliver lifestyle counselling that matches the patients´ needs.

Therefore, the overall aim of this study was to explore patients´ and CHNs experiences of lifestyle counselling in primary care to support healthier lifestyle habits.

## Material and methods

### Design

To explore patients’ and CHNs’ experiences of lifestyle counselling we used a qualitative design and performed a content analysis with an inductive approach [19, 20].

### Participants and recruitment

We used a purposive sampling from the population of individuals who completed the one-year lifestyle programme in 2015. To gather as rich data as possible, we included participants of different sex, age, and diagnoses. Secondly, inclusion criteria were being fluent in Swedish and having participated in all five counselling sessions with CHNs. Eligible patients were contacted in May 2018 by the CHNs and asked to consent to an interview with the first author, LL (physiotherapist, previously employed at the primary care center, PhD student). A total of 16 patients were interviewed: eight women and eight men, age 51–75 years. Nine patients had hypertension, six patients had T2DM, and one patient had impaired glucose tolerance. A more detailed description has been presented earlier [21].

To enrich the data, we interviewed three of the four CHNs who had performed the counselling in 2015; the fourth CHN had ended her employment at the time of the interview. All CHNs had over ten years’ experience of lifestyle counselling and in-depth knowledge about diabetes care, the metabolic syndrome, and motivational interviewing (MI).

This study was approved by the local ethics committee in Uppsala (DNR 2014/497/1) [22]. Quotations from the CHNs are presented without referring to any specific CHN to ensure their anonymity. The study was performed according to good research practices and the Declaration of Helsinki [23] and included provision of written and oral information to interested people, voluntary participation, and secure data management. All informants provided informed written consent.

### The one-year lifestyle counselling programme

The lifestyle programme comprised five individualized, face-to-face appointments, with a CHN for one year with focus on counselling to enhance patient’s lifestyle habits and has been described earlier by Lönnberg et al. [16]. See Table 1 for the overall content of the one-year lifestyle programme.

**Table 1:** Content of the five individual counselling sessions in the one-year lifestyle programme

	**Baseline appointment**	**Appointment at 3, 6, 9 months**	**One-year appointment**
Content of the counselling	Individual counselling (discussing prerequisites and motivation for lifestyle change, setting behavioural and treatment goals, verbal and written information on lifestyle habits in relation to diagnose)	Individual counselling (follow up by discussing content in diaries for food or physical activity, evaluation of goal achievement, exploring barriers, setting new goals, providing information regarding lifestyle in relation to diagnose)	Individual counselling (same as previous appointments including summarizing of experiences and results of the past 12 months, setting new goals)
Measures	Fasting blood sample Anthropometric measurements Submaximal oxygen uptake test (bicycle) Questionnaire	Blood pressure Waist circumference	Fasting blood sample Anthropometric measurements Submaximal oxygen uptake test (bicycle) Questionnaire

Motivational interviewing was used to explore and strengthen the patient’s ability to make lifestyle changes. Motivational interviewing was first described by Millner and Rollnick [24] and is a person-centerd method by which the person’s own choices are explored and decisions about goals and actions are made in dialogue between the patient and health-care professionals. All CHNs were trained in motivational interviewing and had, at minimum, attended a one-week fulltime education in motivational interviewing. The training focused on enhancing practical skills to support and strengthen the patient’s ability to perform lifestyle change.

A self-administered questionnaire was used to obtain information about current lifestyle habits, and results of anthropometric measurements and blood sampling were discussed with the patient. Both behavioural and treatment goals were set in dialogue and had to be accepted by the patient. Patients were also invited to participate in evening group sessions that covered different lifestyle related topics.

### Data collection

All individual interviews of patients were conducted by LL and took place between July and November 2018 at the primary care unit. In total, 16 patients were interviewed. After 12 interviews, the information started to repeat and after four more interviews, the information collected was considered rich enough (i.e. there was a redundance of information). The interview of CHNs was performed in May 2020 by ÅR (physiotherapist and researcher) as a focus group interview. The individual interviews lasted between 30–45 min and the focus group 90 min. The interviews were audio recorded and transcribed verbatim by LL.

The semi-structured interviews of patients and CHNs followed an interview guide prepared by LL and ÅR (see Table 2) with two main questions in focus: (1) What were the participants’ experiences of lifestyle change? and (2) What were the participants’ experiences of lifestyle counselling?. The interview guide was piloted with the first two participants and was found appropriate.

**Table 2:** Interview guide with questions used to explore patients’ and community health nurses’ experiences of lifestyle counselling

Background information	Previous experiences of counselling? (P, CHN) Are you receiving or giving counselling today? (P, CHN) What kind of education do you have that is relevant to providing lifestyle counselling, e.g. diabetes care, metabolic syndrome, MI? (CHN)
About lifestyle habits	Which habits were addressed? (P, CHN) Who prioritized which habits to address? (P, CHN) Who sets the goals? (P, CHN)
About counselling	What components of counselling do you find helpful? (P, CHN) What motivates you to change your lifestyle habits? (P) How do you act to enhance motivation? (CHN) What are the easy parts of providing counselling, and what is more difficult? (CHN)
About maintenance	What do you need to maintain a healthier lifestyle? (P) How do you prepare the patient to maintain a healthier lifestyle? (CHN)
About the supervisor role	How do you want the CHN to guide you? (P) How do you see your role as supervisor – ‘carrot or stick’? (CHN) When the patient doesn’t adhere to the advice – what do you do? (CHN)
Final comments	Is there something else you want to add? (P, CHN)

Every question was followed up with prompts such as, ‘Can you tell me more?’ or ‘Can you give me some more examples?’. *P* = question for patients, *CHN* = question for community health nurses, *MI* = motivational interviewing

### Data analysis

To explore the patients’ and CHNs’ experiences of lifestyle counselling, a qualitative content analysis was performed [19, 20].

**Fig.1:**
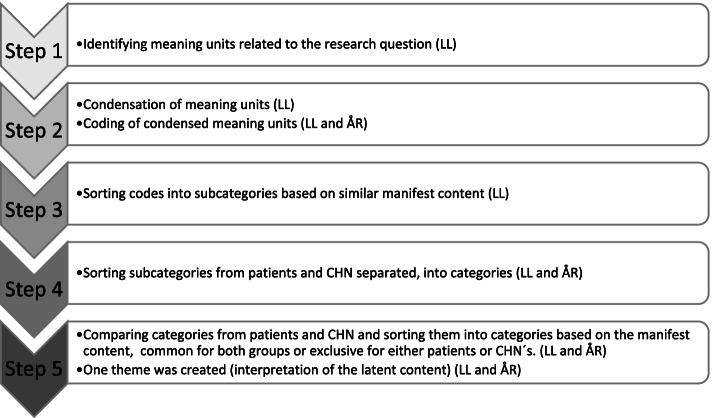
Description of the different steps in the qualitative content analysis process. (LL –Lena Lönnberg, ÅR – Åsa Revenäs)

All interviews were reread several times to obtain an overview and an overall sense of the material. The different steps in the analysis process are described in Fig. 1.

An example of the analysis is presented in Table 3.

**Table 3:** Example from the analysis to transform meaning units to condensed meaning units, codes, subcategories and categories

Meaning unit	Condensed meaning unit	Code	Subcategory	Category
‘… yes, I was more persistent, I would think. With the measurements you become more – ok let’s do this’ *(female, hypertension, 67 years)*	Was more persistent with physical activity when followed up with measurements	Measurements motivate a change in lifestyle	Measurements increase motivation and help to clarify current condition	Measurements and goal setting are valuable, but knowledge is required
‘…yes, it feels safe. To have the long-term blood sugar checked… and at the same time being able to discuss the situation overall, that´s positive.” *(female, T2DM, 75 years)*	Feels safe when long-term blood sugar is measured. Positive to discuss the situation	Feeling safe with repeated measurements and discussions	Measurements increase motivation and help to clarify current condition	Measurements and goal setting are valuable, but knowledge is required

### Trustworthiness

The interviews were transcribed by LL shortly after they had been performed. The transcripts were checked against the audio files by LL on two occasions, and for the two test interviews, also by ÅR. The interview with the CHNs was performed by ÅR. The first two interviews were coded by both researchers (LL and ÅR) and then discussed, to improve reliability of the coding. The categorization was discussed several times by LL and ÅR to increase coherence and to ensure that the subcategories and categories were independent and able to be distinguished from each other. Reflexive memos of the discussions between LL and AR were maintained by LL during the process of data collection and analysis. This was done to maintain an awareness of personal biases or judgements during the process of analysis.

The results were also scrutinized by two independent researchers experienced in qualitative research but not previously involved in the project, on two different occasions. One of the authors (LL) used to work as a physiotherapist at the primary care center. There was no patient-provider relationship with anyone of the interviewed patients, but LL had a relationship with the CHN´s as a former co-worker. This implies that LL had to combine the roles of being both an insider and an outsider at the same time. To balance this, the interview with the CHNs was performed by ÅR (physiotherapist, researcher in the field of digital support for improved health, with special focus on behaviour support for physical activity), who had not been involved in the lifestyle program. During the analysis process all codes, subcategories and categories were scrutinized by both authors, checking them with the original data material at several occasions throughout the analysis process. Mattias Damberg (MD) used to work as a physician at the primary care center. MD was responsible for the development and design of the lifestyle program.

## Results

The results are presented below in the following structure. First, the theme is presented, and the latent content of the interviews is interpreted. Next, the description of each category, which reflects the manifest content, starts with a short summary of the main content and is followed by a description of some of the subcategories with quotations. When similar concepts were expressed by both patients and CHNs, the term informants is used; otherwise the quotes are identified as coming from patients or CHNs.

### Lifestyle counselling – a long-term commitment based on a partnership

The theme emphasizes that lifestyle counselling is a task for two people and that the patient and CHN worked in partnership to set treatment and behavioural goals. The theme incorporates five categories that describe how the participants experience lifestyle counselling. The informants noted that this partnership should be based on mutual respect, recognizing the patient as the expert on his/her current life situation and that both parties had to engage in the process of lifestyle change. A summary of the results with theme, categories and subcategories is presented in Table 4.

**Table 4:** Theme, categories and subcategories that describe the participants ‘experiences of lifestyle counselling

*Theme*	*Lifestyle counselling – a long-term commitment based on a partnership*				
**Categories**	**Collaboration should be based on respect and mutual interest**	**Counselling should facilitate understanding of illness and lifestyle habits**	**Measurements and goal setting are valuable, but knowledge is required**	**Long-term support after the end of the lifestyle programme is important**	**The care unit should support counselling for lifestyle change**
Subcategories	A good alliance enables a trusting dialogue and an equal partnership	Counselling should provide and/or increase knowledge about lifestyle habits	Measurements increase motivation and clarify the current condition	Recurrent counselling is needed to maintain lifestyle change (P)	Different modes of delivery should be used for counselling
	Dialogue provides confirmation and exploration of the patient’s need for support	Counselling should provide and/or increase knowledge about illness	Goal setting is important for increasing motivation (P)	It can be burdensome to monitor illness by myself (P)	The lifestyle programme should include a structure for the delivery of counselling (CHN)
	Recurrent dialogue motivates change and builds relationships	Counselling should provide/increase knowledge about how lifestyle actions can affect illness	Measurements can be difficult to interpret (P)	Support should focus on maintenance and positive changes (CHN)	Continuous education is needed for health-care professionals to maintain and improve their counselling skills (CHN)
	External control by the nurse supports lifestyle changes (P)	Both patients and CHN should be aware that medication can affect motivation (P)	Patients need support to set achievable goals (CHN)		
			Treatment goals should be consistent with the guidelines (CHN)		

CHN = subcategories expressed only by nurses, P = subcategories expressed only by patients. Subcategories without (CHN) or (P) refer to experiences of both nurses and patients

### Collaboration should be based on respect and mutual interest

Lifestyle counselling was viewed as involving two parties who together elaborated on the patient’s need of support to make lifestyle changes (Table 4).

The informants noted that the alliance and dialogue were important to the successful design of lifestyle counselling programme. The patients voiced that they were more willing to talk about difficulties and setbacks when they had a trusting relationship with the CHN. They also noted that confirmation of their current healthy habits was helpful and strengthened their sense of self-efficacy.

*“…It’s very important who gives the counselling — that it is the right person, and it was! It was the right person, someone who is soft, can listen and who comes with suggestions. And someone to discuss different matters with…” (woman, hypertension)*

The CHNs also considered the recurrent contact as an important tool for building a relationship with the patient. Understanding and exploring each patient’s living circumstances, the type of support he/she needs, and the possible barriers were noted as essential to tailoring the support for patients to adopt a healthier lifestyle.

*“and when you meet each patient so often, you build up a trust and they could say – this didn´t work in another way than when he or she perhaps met their physician…” (CHN)*

However, some patients voiced that they wanted stricter recommendations about how lifestyle habits should be altered and expressed a need for a ‘control function’ involving a firmer attitude by the CHN.

### Counselling should facilitate understanding of illness and lifestyle habits

This category demonstrates the importance of providing information and increasing knowledge of the disease, lifestyle habits, effects of lifestyle actions on the condition, and possible need for medication (Table 4).

The informants noted that increased knowledge about how lifestyle habits affect the disease itself, and how the individual could cope with his/her condition were important. Even though the CHNs acted as the provider of information and patients as the ‘receiver’, the informants identified themselves as co-creators of the process of increasing the patient’s knowledge. The CHNs commented that providing knowledge about both the disease and its relationship with lifestyle habits was essential to deepening the patients’ understanding of how their own actions can affect their condition.

*“…You have to point out all the advantages of what happens with enhanced measurements. And you can also point out what will happen if the values go in the wrong direction, complications, and so on.” CHN).*

The patients considered knowledge about how medication can affect both the condition and motivation for lifestyle changes as important. Fear of having to take medication was motivational for patients to make healthier choices. On the other hand, one patient noted that medication could imply a ‘short cut’, that is, taking medication instead of having to change lifestyle, identifying a fear that *taking* medication could impair motivation for lifestyle.

*“…I’m grateful for not having to take medication. If I would have had to take glucose-lowering treatment, I could have cheated…” (woman, T2DM)*

### Measurements and goal setting are valuable, but knowledge is required

The informants considered repeated anthropometric measurements, blood sampling and goal setting as motivating and important elements in lifestyle counselling. However, the findings also revealed difficulties in interpreting these measures and setting realistic goals (Table 4).

The informants stated that repeated measurements enhanced the patient’s motivation and provided opportunities to make stepwise changes to reach the set treatment goal. However, the informants focused on different aspects of the value of measurements and goal setting.

The patients noted that repeated measurements implied a sense of safety by clarifying their current condition and making it easier to identify any effects from lifestyle changes on blood pressure or blood glucose. Reaching a set treatment goal was motivating as this confirmed the patient’s adoption of a healthier lifestyle. Some patients noted difficulties in understanding the results of measurements or that interpretation of the results could be difficult and therefor requested more information.

*“…After the consultation I received a letter that told me that the results were ‘within normal reference value’. But I want to know if it was in the upper part or lower, or somewhere in between. I never got the exact figures…” (man, T2DM).*

From the CHNs’ point of view, goal setting was difficult to do by the patients themselves even though goals must be individualized, realistic, and accepted by the patient. Goals were set in dialogue with the patient after exploring their preferences and current motivation for lifestyle change, however they also strived for treatment goals based on relevant guidelines.

*“Goals are set together with the patient. Test results and ‘normal values’ are indicative. It also depends on age and other conditions that influence what the treatment goal should be. (CHN).*

### Long-term support after the end of the lifestyle programme is important

The informants expressed a need for further individualized contact to maintain the changed lifestyle habits after the end of the lifestyle programme both regarding counselling to support maintenance of lifestyle habits and to resolve questions regarding illness and medication. (Table 4).

Patients commented that recurrent contact after the end of the lifestyle programme was important for maintenance. The need for support in terms of frequency varied, and they noted that ‘booster sessions’ could be of value as e.g. the regular follow-ups for patients with T2DM. Patients with hypertension noted that this aspect was missing and that the lack of counselling after the end of the lifestyle programme left them alone to both check their blood pressure and to know when a new prescription of medication was needed. The patients experienced that it was their responsibility to make contact.

*“No one has contacted me… It is me who makes a phone call and request a new prescription to a voice mail once a year” (man, hypertension)*

The CHNs commented that their role was to support maintenance of enhanced lifestyle habits after the programme and focused on setting reachable goals and supporting lifestyle changes that could be sustained over time.

### The care unit should support counselling for lifestyle change

This category includes understanding about how different aspects of the organization of the care unit were important to support lifestyle counselling. The structure should support counselling according to patients’ needs and include continuous education of health-care professionals (Table 4).

The informants noted the value of using different modes for delivering counselling; face to face, by phone or internet, individual or group based. They also voiced that being able to communicate via mobile phone, SMS, or email facilitated communication.

The CHNs found that the structure of the lifestyle programme delivered with a planned and organized lifestyle counselling was useful. For example, the questionnaire about current lifestyle habits acted as a starting point for counselling – as a ‘door-opener’ to a dialogue about all lifestyle habits.

*“…It felt quite natural when we looked at the answers in the questionnaire; then it was ok to discuss everything…” (CHN)*

According to the CNHs, the involvement of the entire primary care center was also important to facilitate lifestyle counselling. Patients could obtain information and support from other professionals at the primary care unit, which reinforced counselling from the CHNs.

## Discussion and conclusion

### Discussion

The overarching theme identifies that the essence of the informants’ experiences was that counselling should be based on partnership. Both patients and CHNs emphasized that their alliance was the cornerstone for addressing a personal matter such as lifestyle habits. Both parties also noted that recurrent appointments over time allowed them to build a trusting relationship that allowed them to discuss both behaviour changes that were easy as well as lifestyle matters that were more challenging. The informants recognized that counselling is a joint process that acknowledges both the patients and CHNs as co-creators of the process to explore the actions needed to change lifestyle habits to improve the patient’s health. The partnership was considered to be essential to ensure a trusting relationship in which success, barriers, and setbacks could be elaborated. Increased knowledge, repeated measurements, and long-term support were also considered to be central elements to reaching the set goals.

The CHNs noted that recurrent appointments with the same patient enabled them to take a step back and encourage the patient to make his/her own decisions and experiences. This is consistent with the MI technique, in which a key feature is noting the patient’s ambivalence, emphathetic and reflecting listening, strengthen self-efficacy and thereby enhance maintenance [24]. Although MI is commonly used in primary care for health-related behaviour change, research demonstrates an inconsistency in its outcomes. For example, a review article by Morton et al. examined the evidence base for MI intervention to achieve behaviour change in non-clinical populations in primary care settings. Only about 50% of the included studies reported positive outcomes relating to behaviour change. The review also revealed an inconsistency in the descriptions and intervention components of the MI, which made it difficult to identify their effect [25]. On the other hand, a meta-analysis that synthesized findings from 12 randomized controlled studies of MI interventions for health behaviour outcomes in primary care populations found that MI appeared to be useful for outcomes related to weight loss, blood pressure, and substance use [26]. The results of our previous study of reduced CV risk after participation in the one-year lifestyle programme [17] are consistent with the conclusions of this meta-analysis [26].

The counselling in our study involved several different BCTs [12] to enhance motivation and support maintenance of lifestyle change. For example, repeated measurements, goal setting, and use of diaries for self-monitoring of physical activity and food intake were recognized as useful tools. This is consistent with the findings of a meta-regression analysis by Michie et al. in which interventions that combined self-monitoring with at least one other BCT were found to be significantly more effective than the other interventions [27].

Goal setting in our study comprised both treatment goals and behavioural goals. However, both patients and CHNs seemed to use the measurements of metabolic risk factors to evaluate behaviour change. This implies that the treatment goal was more equivalent to an ‘outcome goal’ as defined by Michie et al [12]. For example, if the patient reached the set blood pressure goal, this was acknowledged as a successful change of lifestyle habit even though this might not have been the case. On the other hand, a successful change of physical activity might not result in a lowered blood pressure for an individual patient. Perhaps the use of other BCTs, such as modelling the behaviour, reviews of behavioural goals, and action planning including implementation of intentions, could support the patients’ ability to enhance lifestyle habits even better. Evaluating the behavioural goal along with an outcome goal might also stop patients from seeing medication as a short-cut or a way of ‘cheating’ and instead be motivational to lifestyle changes.

The informants voiced that a trustful relationship was a cornerstone in the process of making lifestyle changes. This alliance built a solid base were the patient and CHN, together, could explore the different steps that needed to be taken by the patient; where patients could feel secure/safe in sharing their difficulties and unhealthy habits without being ashamed, and where the CHN could listen without judging. The alliance with the CHN was thereby perceived as strengthening the patient’s self-efficacy by exploring barriers, making stepwise changes, and confirming healthier lifestyle habits. Self-efficacy describes a person’s perception of his/her capacity to take action and persist in that action [28]. It is a common concept in several learning and behaviour theories such as the transtheoretical model of behaviour change [29] and social cognitive theory [30]. Self-efficacy has been shown to be a key component of the success at organizing and implementing changes in both physical activity and diet [11, 31]. It has also been demonstrated that patients with high self-efficacy are more likely to adhere to self-care in their management of e.g. hypertension [32]. Hence, one key component of lifestyle counselling is to strengthen self-efficacy.

In our study, patients with hypertension commented that they felt they were left alone to follow up their condition after the end of the lifestyle programme. This contrasts with existing guidelines for hypertension which includes a yearly follow up on blood pressure, current lifestyle habits and prescription of blood pressure lowering medication, if indicated. Patients with hypertension experienced that they did not receive attention from their healthcare providers unless they made contact themselves. This was articulated as being burdensome by the patients. When the set treatment goal is reached, a yearly follow-up with a CHN or family physician is recommended by international, Swedish, and local guidelines for the management of hypertension [4, 33]. The patients expressed a desire for a more structured long-term support, a short contact by phone or email to confirm whether their current blood pressure was within the target range. This short contact may also provide an opportunity to follow up on behaviour changes. Previous studies of the implementation of mobile phone or web-based interventions have reported positive effects on blood pressure control and may provide a suitable method for meeting the patients’ requests for feedback [34, 35].

The CHNs noted that it is important to have a structure within the primary care unit that supports the lifestyle programme. This included making time available, supporting continuing education of CHNs and increasing understanding of the importance of lifestyle habits among all health care professionals. Having a solid foundation at the primary care unit supported the CHNs as they strive to enhance the care of patients at high risk of CVD.

This qualitative study has several strengths. First, we found that the chosen informants were suitable to provide an in-depth experience of the lifestyle programme as they comprised of actual programme participants and CHNs involved in the delivery of the lifestyle programme. By collecting data from both parties, we were able to explore their experiences of lifestyle counselling and to obtain an in-depth understanding about which components of the lifestyle programme are essential. Second, trustworthiness has been addressed in several ways in our study: LL performed all patient interviews; ÅR performed the focus group interview of CHNs; and two independent researchers scrutinized the results on two different occasions. Third, we have described the methods and results in detail for transparency and to provide the reader the opportunity to judge the trustworthiness and transferability of the results.

The study also has some limitations. First, we did not interview patients who dropped out of the lifestyle programme. Patients who dropped out of the lifestyle programme could have had different experiences or viewpoints from those interviewed for this study. The rationale for this is that this study is a part of an evaluation of the one-year lifestyle programme [16, 17, 21], and we therefore chose to interview patients who attended all five counselling sessions. Second, the interviews took place three years after the patients participated in the programme, which may have imposed a recall bias, although the elapsed time may have provided a better perspective on the lifestyle programme. Finally, the primary care unit where the lifestyle programme was performed treats patients from high socio-economic circumstances, as indicated by a low Care Need Index [36], which may hamper transferability.

### Conclusion

The results of this study are consistent with previous knowledge about successful delivery of lifestyle counselling in the context of Swedish primary health care. The informants declared that counselling must be based on partnership, which they characterized as being based on mutual respect and the recognition of the patient as expert on his/her current life situation, and that both parties had to engage in the process of lifestyle change. A trusting relationship, increased knowledge about lifestyle habits and disease, repeated measurements, recurrent counselling with the same CHN, and the need for the lifestyle programme to be structured within the primary care unit were experienced as core elements to increase patients ability for self-management.

### Practice implications

This study underlines that a structured lifestyle program with five counselling sessions within primary care is experienced as helpful and feasible by both patients at high risk of CVD and CHNs. The findings underline that partnership and continuity, i.e. meeting the same health care provider is crucial to strengthen self-efficacy regarding enhanced lifestyle habits. In addition, long-term follow-up was expressed as an important factor to support maintenance and finally, the management of the care unit needs to support the delivery of a structured lifestyle program.

## Data Availability

The datasets generated and / or analysed during the current study are not publicly available because they contain confidential and sensitive data. Anonymously labelled data are available from the corresponding author on reasonable request.

## Abbreviations

BCT: Behavioural change technique
CHN: Community health nurse
CV: Cardiovascular
CVD: Cardiovascular disease
MI: Motivational interviewing
